# Vacuum-assisted breast biopsy in close proximity to the skin: a case report

**DOI:** 10.1186/1752-1947-2-165

**Published:** 2008-05-18

**Authors:** Flora Zagouri, Theodoros N Sergentanis, Dimitra Koulocheri, Georgia Giannakopoulou, Aphrodite Nonni, Dimitrios Dardamanis, Nikolaos V Michalopoulos, Ioannis Flessas, John Bramis, George C Zografos

**Affiliations:** 1 Breast Unit, 1st Department of Propaedeutic Surgery, Hippokratio Hospital, School of Medicine, University of Athens, Greece; 2 First Department of Pathology, School of Medicine, University of Athens, Greece

## Abstract

**Introduction:**

Vacuum-assisted breast biopsy is a minimally invasive technique used increasingly for the assessment of mammographically detected, non-palpable breast lesions. The effectiveness of vacuum-assisted breast biopsy has been demonstrated on lesions both with and without microcalcifications. Given that the position of the lesion represents a major factor in stereotactic vacuum-assisted breast biopsy, targeting lesions in close proximity to the skin (superficial lesions) has been described as a problematic issue.

**Case presentation:**

A 53-year-old woman presented with a newly developed, non-palpable lesion in her left breast. The lesion consisted of widely spread microcalcifications located approximately 5 mm from the skin. The lesion was isoechoic on ultrasound examination. Vacuum-assisted breast biopsy was scheduled (on the Fischer's table, using 11-gauge probes, under local anaesthesia). The vacuum-assisted breast biopsy probe was inserted antidiametrically into the breast, the probe reached the lesion and effort was made to excise the microcalcifications. As only a small proportion of the microcalcifications were excised an accurate diagnosis could not be expected. However, with the probe having entered the breast antidiametrically, the probe tip underlying the skin could be palpated. Following the palpation of the tip, the exact point was marked by a pen, the probe was removed and the patient was transferred to the surgery room to have the remaining lesion removed by a spindle-form excision under local anaesthesia. The mammogram of the removed specimen confirmed the total excision of the suspicious microcalcifications.

**Conclusion:**

Isoechoic superficial lesions can be localized with a hook-wire and open breast biopsy under general or local anaesthesia can be performed. However, vacuum-assisted breast biopsy might offer an alternative solution and serve as an alternative approach to localize the lesion. The clinical significance of the present exploratory effort remains to be assessed in the future.

## Introduction

Vacuum-assisted breast biopsy (VABB) is a minimally invasive technique used increasingly for the assessment of mammographically detected, non-palpable breast lesions [1,2]. The effectiveness of VABB has been demonstrated on lesions both with and without microcalcifications [3,4]. The sensitivity, specificity and fast performance of the method have contributed to its gradual establishment in the workup of suspicious breast lesions [1,2,5].

The contraindications of the method are few, but they do exist. Stereotactic VABB is contraindicated for patients who cannot cooperate with the procedure or who have a bleeding diathesis. Inability to tolerate local anaesthesia is a further rare contraindication. Most importantly, stereotactic VABB is contraindicated for lesions that cannot be targeted (that is, cannot be definitively identified on stereotactic images) or cannot be included on the images (due to extreme posterior positions, for example) [6].

Given that the position of the lesion represents a major factor in stereotactic VABB, targeting lesions in close proximity to the skin (superficial lesions) has been described as a problematic issue [6,7]. In this case report, an alternative procedure has been adopted on an isoechoic, superficial non-palpable breast lesion (BI-RADS 4A) located 5 mm from the skin.

## Case presentation

A 53-year-old woman came to our Breast Unit for her annual mammogram. A newly developed, non-palpable lesion was present in her left breast. The lesion consisted of widely spread microcalcifications located approximately 5 mm from the skin. Axillary lymph nodes of small size were detected on the mammogram. The lesion was isoechoic at ultrasound examination.

The woman had risk factors for breast cancer, with a positive family history for breast cancer (mother with post-menopausal breast carcinoma). Her body mass index was 24 and she was a housewife. Her age at menarche was 11 years and her age at menopause was 47 years. She reported two induced abortions, one full-term pregnancy, 2 months duration of lactation and intake of oestrogen for 3 years (between the ages of 21 to 24 years old). The patient reported no family history for cancer of the ovary or prostate.

The radiologist characterized the suspicious lesion in the upper outer quadrant of the left breast as BI-RADS 4A (lesion with a low suspicion for malignancy) and the breast density as category 3 (heterogeneously dense, classified according to the American College of Radiology breast imaging and reporting data system categories). Subsequently, a VABB was scheduled. VABB was performed on a digital prone table (Mammotest, Fischer Imaging, Denver, CO, USA) using 11-gauge Mammotome vacuum probes, under local anaesthesia.

**Figure 1:**
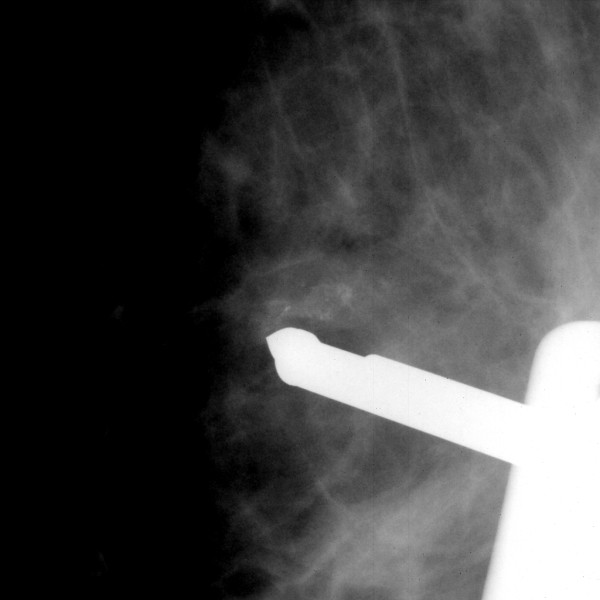
The suspicious lesion and the vacuum-assisted breast biopsy probe underneath the skin.

The VABB probe was inserted antidiametrically, the probe reached the lesion and effort was made to excise microcalcifications (Figure 1). The X-ray of the specimens revealed that only a small proportion of microcalcifications were excised and thus an accurate diagnosis could not be expected to be established. However, with the probe having entered the breast antidiametrically, the probe tip underlying the skin could be palpated.

**Figure 2:**
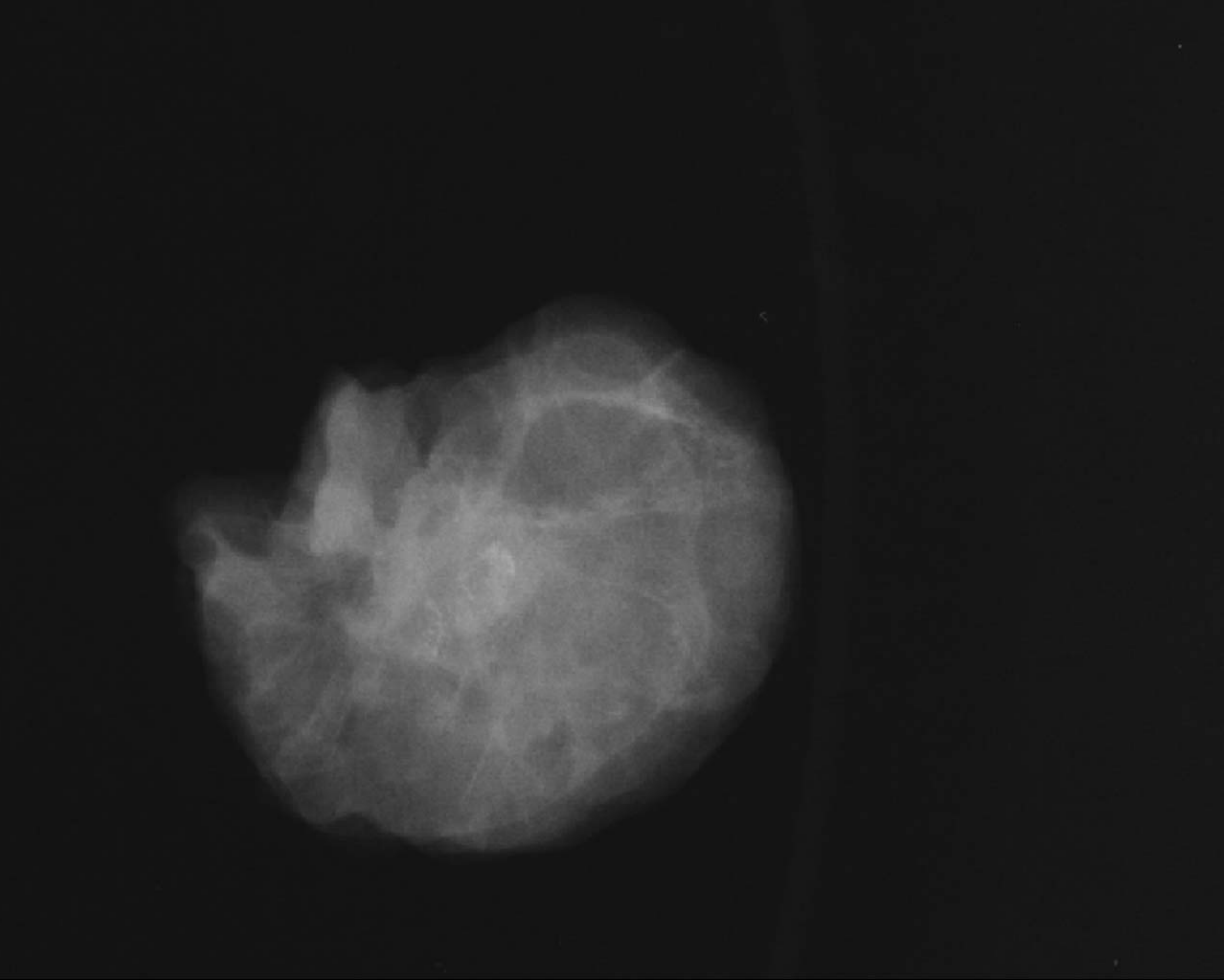
Specimen derived after the spindle-form excision of the whole suspicious lesion.

Following the palpation of the tip, the exact point was marked by a pen, the probe was removed, the breast was decompressed and the woman returned to the supine position. The patient was then transferred to the surgery room and the remaining suspicious lesion was removed by a spindleform excision under local anaesthesia. Subsequently, the mammogram of the removed specimen confirmed the total excision of the suspicious microcalcifications (Figure 2).

According to the pathological examination, the main diagnosis for the lesion was a benign condition, sclerosing adenosis.

## Discussion

Targeting non-palpable superficial lesions is an unusual problem faced in the context of VABB. Indeed, when targeting an extremely superficial lesion, part of the collecting area (bowl) of the probe might not have fully entered the breast, and thus vacuum cannot be achieved. Advancing the probe deeper and sampling the lesion with the probe past the centre of the lesion has been proposed. Other proposed approaches are to raise a generous wheal of local anaesthetic in the skin so as to increase the depth of the lesion, or even to use a skin hook to pull tissue over the probe entry site [6].

In this case, an alternative procedure has been adopted. An isoechoic superficial breast lesion can be localized with a hook-wire and open breast biopsy performed under general or local anaesthesia. Interestingly, VABB might still have a role in offering an alternative solution. Indeed, the VABB probe (antidiametrically inside the breast) was used in this case as an alternative way to localize the lesion.

In order to evaluate the clinical significance of this alternative intervention, it should be kept in mind that VABB has been shown to be less painful than a biopsy with hook-wire localization [8]. Additionally, the procedures mentioned above have not been systematically explored, and the pain or complications caused by them remain unknown, especially when using a skin hook to pull the tissue. Additionally, it should be noted that VABB has not been approved for this procedure, whereas localization with a hook-wire is approved; the latter needs no incision and the needle is smaller. Moreover, other options such as 14G probe or ultrasound-guided VABB exist and could alternatively be used.

This report represents only a preliminary effort of an exploratory nature; it does indicate, however, the need for discussion about difficult positions in VABB. Previously, positions difficult to sample have remained a somewhat underinvestigated issue.

## Conclusion

To the best of the authors' knowledge, this is the first case in the international literature where VABB was used in an alternative way to sample and localize a superficial breast lesion. VABB potentially offers numerous possibilities, which remain to be assessed in the future.

## Competing interests

The authors declare that they have no competing interests.

## Authors' contributions

FZ conceived the idea of the study and wrote the manuscript. TNS interpreted the case findings with respect to the international literature and wrote the manuscript. DK and GG assigned the BI-RADS category and assisted in VABB; both helped in editing the manuscript. AN made the pathological diagnosis. DD and NVM performed VABB and participated in the evaluation of the case findings. IF contributed to the review of the literature and assisted in VABB. JB revised the manuscript for important intellectual content. GCZ supervised and performed VABB, interpreted the findings, revised critically the manuscript for important intellectual content and gave final approval of the version to be published. All authors read and approved the final manuscript.

## Consent

Written informed consent was obtained from the patient for publication of this case report and accompanying images. A copy of the written consent is available for review by the Editor-in-Chief of this journal.
